# Tuberculosis screening and care delivery in pregnant and postpartum persons living with HIV in Botswana

**DOI:** 10.1371/journal.pgph.0005784

**Published:** 2026-01-12

**Authors:** Melanie M. Dubois, Mackenzie Powell, Sara Schenkel, Samuel Kgole, Gosego Masasa, Martha Ngwaca, Boitshepo Phale, Ame Diphoko, Coulson Kgathi, Gaerolwe Masheto, Joseph Makhema, Topo Makhondo, Chidzani Mbenge, Tanvi Sharma, Radhika Sundararajan, Jyoti Mathad, Chawangwa Modongo, Lisa M. Butler, Daniel Fitzgerald, Kathleen M. Powis

**Affiliations:** 1 Division of Pediatric Infectious Diseases, Weill Cornell Medicine, New York, New York, United States of America; 2 Department of Pediatric Global Health, Massachusetts General Hospital, Boston, Massachusetts, United States of America; 3 Department of Public Health Sciences, Queen’s University School of Medicine, Kingston, Ontario, Canada; 4 Botswana Harvard Health Partnership, Gaborone, Botswana; 5 National TB Program, Ministry of Health, Gaborone, Botswana; 6 Division of Infectious Diseases, Boston Children’s Hospital, Boston, Massachusetts, United States of America; 7 Center for Global Health, Weill Cornell Medicine, New York, New York, United States of America; 8 Victus Global Botswana Organization, Gaborone, Botswana; 9 Division of Internal Medicine and Pediatrics, Massachusetts General Hospital, Boston, Massachusetts, United States of America; 10 Department of Immunology and Infectious Diseases, Harvard T.H. Chan School of Public Health, Boston, Massachusetts, United States of America; Burnet Institute, AUSTRALIA

## Abstract

Tuberculosis (TB) disease during pregnancy, particularly in persons living with HIV (PLHIV), is associated with poor maternal and neonatal outcomes. There are barriers to TB screening and care for PLHIV in the pregnant and postpartum period given atypical clinical presentation and service delivery challenges. Our objective was to understand barriers and facilitators to TB screening and care delivery during routine antenatal and postpartum care in Botswana government health centers among PLHIV and healthcare providers. In this mixed methods study conducted between April 2022 and November 2023, quantitative data was collected from PLHIV on frequency of TB screening at their antenatal and postpartum government health center visits. At a 2-month postpartum study visit, PLHIV were screened for TB symptoms by study staff, using the World Health Organization TB symptom screen, and referred to their local government health center if positive. Qualitative data was obtained from semi-structured interviews with maternal participants, who screened positive for TB and were referred to government clinics for evaluation, and medical staff from referral clinics. Ninety-five pregnant and postpartum PLHIV consented to study participation. Of the 95 pregnant PLHIV, 9 participants were referred to their local government health facility after screening positive for TB symptoms; 8 of these participants participated in qualitative interviews, along with 9 medical staff. TB screening and care facilitators and barriers for maternal and medical staff participants were identified according to the environmental and population level guided by Andersen’s Behavioral Model. Facilitators to TB screening and care included HIV diagnosis, pregnancy, and proximity to clinics. Barriers included challenges with diagnostic workup, resource limitations, and knowledge gaps. Our findings highlight challenges of TB screening and care for pregnant and postpartum PLHIV. Further studies are needed to evaluate interventions to improve and support TB screening and care delivery for pregnant and postpartum PLHIV.

## Introduction

Tuberculosis (TB) is a major cause of maternal and child morbidity and mortality worldwide [1,2]. In 2023, an estimated 10.8 million people developed TB, of which 33% were adult women [3]. Models estimate that the burden of TB in pregnancy is substantial, with about 200,000 cases of TB disease in pregnant women annually, with more than 70% of TB cases occurring in the World Health Organization (WHO) African and South East Asian regions [2]. Yet there remains lack of global data on the specific burden of TB among pregnant and postpartum persons, with maternal TB surveillance as a global priority [4]. TB disease during pregnancy, particularly among persons living with HIV (PLHIV), is associated with poor maternal and neonatal outcomes [1,5–9]. Maternal risk of TB disease increases late in pregnancy and remains elevated in the postpartum period, due to immunological changes late in pregnancy and postpartum [1,10–12]. The WHO recommends TB symptom screening for all pregnant persons living in high prevalence TB settings as part of antenatal care [13,14]. Specific for PLHIV, the WHO recommends TB screening at each health visit, including the pregnant and postpartum period [14,15]. In theory, maternal-child health (MCH) services for pregnant and postpartum PLHIV can provide a unique opportunity for intensified TB case finding [5]. However, in practice, studies to date have highlighted challenges of TB screening and care delivery for PLHIV during the pregnant/postpartum period in high HIV/TB burden settings, including atypical clinical presentations, limited detection yield with current diagnostics, service delivery challenges, and stigma impacting care seeking behavior and sustained care engagement [16–24]. Several qualitative studies have examined the facilitators and barriers of TB care for PLHIV [25,26], but there are limited qualitative studies on TB screening and care for pregnant/postpartum persons, particularly those living with HIV [27].

In 2023, Botswana had the fourth highest HIV prevalence globally and ranked in the top 30 countries worldwide with high prevalence of both HIV and TB, with an incidence of 244 per 100,000 persons [3,28]. Botswana National TB Programme guidelines recommend intensified case finding for PLHIV, including screening for signs and symptoms of TB at each healthcare encounter [29,30]. These guidelines, in line with the WHO, recommend four screening questions related to cough, fever, night sweats, and weight loss for PLHIV [15]. Botswana guidelines recommend referral for PLHIV screening positive for TB for diagnostic testing with sputum sample and GeneXpert testing at government-run clinics, with services free of charge [30]. Botswana guidelines indicate that adult PLHIV with a negative TB symptom screen are eligible for TB preventative therapy (TPT), which consists of 3 months of once weekly isoniazid and rifapentine (3HP) [30]. In Botswana, TPT is not recommended for pregnant PLHIV, but postpartum PLHIV are eligible for TPT [30,31]. Consistency in applying these recommendations in antenatal and postnatal care clinics, along with barriers and facilitators to TB screening and care delivery during the pregnant/postpartum period for PLHIV, remain unknown in Botswana.

Our objective was to identify barriers and facilitators to the process of TB screening and care delivery during the pregnant/postpartum period, through quantitative data collection on TB screening and qualitative data collection with semi-structured interviews with postpartum PLHIV and medical staff.

## Methods

### Ethics statement

The Institutional Review Boards at the Health Research Development Committee in Botswana, Massachusetts General Hospital, Boston Children’s Hospital, and Weill Cornell Medicine approved the protocol. All individuals participating in the study provided written informed consent prior to study participation.

### Study design, setting, and population

A mixed-methods explanatory sequential study design was employed, which involved collection of quantitative data, with findings informing subsequent qualitative data collection [32]. Quantitative data on TB screening was collected from maternal participants, which refer to pregnant/postpartum PLHIV, enrolled in the study. Qualitative data was collected to further elaborate and explain the quantitative results through semi-structured interviews conducted with maternal participants who screened positive for TB symptoms and were referred to government health centers for further diagnostic evaluation and management, as well as medical staff participants at the clinics where participants were evaluated. This research was carried out according to the Standards for Reporting Qualitative Research (SRQR) and we have adhered to these guidelines and the 21-item checklist for reporting the study’s qualitative results.

The study was conducted at Botswana Harvard Health Partnership (BHP) in Gaborone, Botswana between April 2022 to November 2023. Two study populations were enrolled for this study, including PLHIV enrolled in the FLOURISH study (Following Longitudinal Outcomes to Understand, Report, Intervene and Sustain Health for Infants, Children, Adolescents who are HIV Exposed Uninfected [R61/R33 HD103099; PIs Powis, Jao, Makhema]) and medical staff recruited from antenatal clinics.

Pregnant and postpartum PLHIV participating in the FLOURISH study were recruited to participate in this sub-study. Eligibility for PLHIV required participants to be ≥ 18 years of age, citizens of Botswana, with a pregnancy gestational age of ≥ 22-weeks or up to two months postpartum. Prospective participants had to be able to provide informed consent and have plans to remain in the study area through 6-months postpartum. No clinical care was provided by study staff for sub-study participants.

Eligibility for medical staff included those ≥18 years of age and who provided TB care for pregnant/postpartum PLHIV at government health facilities to which our study participants were referred. We presented the purpose of the study to clinics where participants were referred and subsequently to eligible medical staff who were available. We employed purposive sampling of medical staff to achieve a diverse mix of providers for semi-structured interviews. Eligible medical staff at the clinics where our study participants were referred included internists, medical officers, nurse prescribers, nurses, midwives, and auxiliaries.

### Data collection and analysis

**Fig 1:**
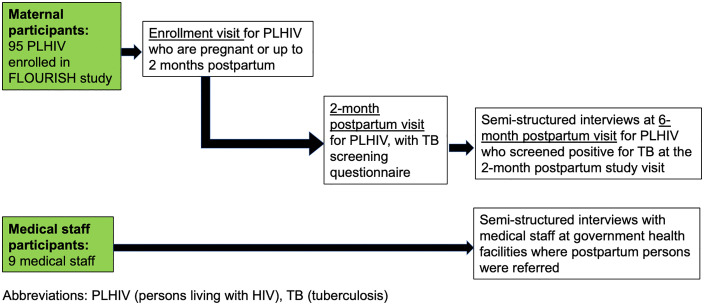
Flowchart of study visits for maternal and medical staff participants.

Quantitative study procedures included up to four study visits with pregnant and postpartum participants (**Fig 1**). As part of the FLOURISH study, there was an enrollment study visit for all PLHIV who consented to study participation and a delivery visit for participants who enrolled while pregnant. For this study, there was an additional 2-month postpartum study visit when all participants were screened for TB symptoms, and a 6-month postpartum study visit only for participants referred following a positive TB screen at the two-month postpartum visit. Demographic and medical history was collected from all PLHIV, including current antiretroviral treatment (ART), history of TB disease, and history of TPT. If a participant’s HIV viral load or CD4 count was reported within the last three months prior to study enrollment, the value was chart abstracted rather than testing the participant. Otherwise, testing for HIV viral load and CD4 was performed at the enrollment study visit. At all study visits, the participant was asked to self-report on whether they were screened for TB at routine health encounters and provide location of screening, including antenatal, infectious disease care clinic (IDCC), postpartum, or other clinic visits, such as outpatient department (OPD), attended since the last study visit.

Maternal participants were screened for TB symptoms of any duration according to the WHO TB symptom screen, including presence of fever, cough, night sweats, or weight loss, at 2-months postpartum by study staff [15]. Participants were asked specifically regarding unexplained weight loss, given that the symptom screen was performed in the postpartum period. Participants were additionally asked about household contacts with TB. If participants screened positive for one or more TB symptoms, the study team referred the participant to their local government clinic for further diagnostic studies and management, where healthcare is provided free of charge. Since this study was specifically designed to understand facilitators and barriers to TB care delivery among pregnant and postpartum PLHIV in government health facilities, diagnosis of TB was not performed in the context of the study and relied on existing standard of care under the Botswana National TB programme.

For maternal participants referred for further TB evaluation to their local government health facility, a study visit was performed at 6 months postpartum, where participants were asked to share their knowledge of TB and participate in a semi-structured interview. The interview guide was structured to learn about the participant’s referral experience, both the clinical aspects and the facilitators and barriers to TB care.

For medical staff participants, there was one study visit to collect data on demographics and TB knowledge, in addition to participation in a semi-structured interview where individuals were asked to share their perspective on TB screening and care for pregnant and postpartum PLHIV (**Fig 1**).

All semi-structured interviews with postpartum PLHIV and medical staff were performed by FLOURISH study nurses trained in the conduct of qualitative interviewing. The interviews were conducted in the participant’s preferred language, either Setswana, the native language of Botswana, or English.

#### Quantitative analysis.

Descriptive statistics were used to evaluate the sociodemographic characteristics of PLHIV and medical staff, including medians and interquartile ranges for continuous variables and counts with frequencies for categorical variables. We calculated the prevalence of PLHIV who reported having been screened for TB at least once during pregnancy through two months postpartum. We calculated the prevalence of PLHIV who screened positive for TB symptoms at the 2-month postpartum visit. Analyses were completed in SAS Version 9.4 and RStudio Version 4.3.2.

#### Qualitative analysis.

Interviews were transcribed and, if needed, translated into English, by GM, SK, MN, and BP. The English transcripts were incorporated into Dedoose software for analysis. The transcripts were coded according to themes based on the Andersen’s Behavior Model of Health Service Use. Andersen’s Behavioral Model (ABM) provides a framework to understand how environmental factors and population characteristics impact health outcomes [33]. These factors can be grouped into ABM domains according to environmental factors, including the health system and the external environment, and population characteristics, including predisposing factors (characteristics intrinsic to the population), enabling factors (resources that encourage health care usage), and perceived need (health care beliefs and values). Codebooks were developed for maternal and medical staff transcripts based on the ABM domains. MMD and MP independently coded all transcripts. To ensure interrater reliability, line-by-line comparisons of the data were performed by MMD and MP, and discrepancies were resolved by consensus. Codes were reviewed to identify themes pertinent to TB screening and care delivery. Summary and descriptive statistics were used to analyze questionnaire responses.

## Results

Between April 2022 and November 2023, 95 PLHIV and nine medical staff were enrolled in the study. Characteristics of PLHIV are presented in **Table 1** (**S1 Data**). The median age of PLHIV was 32 years [IQR 28,37]. All PLHIV were on ART, with median CD4 count of 660 [IQR 509, 835] and 94.7% had an undetectable HIV viral load at enrollment. The majority of PLHIV (97.9%) were receiving dolutegravir (DTG)/lamivudine(3TC) or emtricitabine (FTC)/tenofovir (TDF) for ART, and 86.3% initiated ART prior to conception. Two (2.1%) participants had a prior history of TB. Twenty-two participants (23.2%) reported a history of taking TPT, with 18 (81.8%) reporting having completed TPT.

**Table 1:** Maternal participant characteristics.

Characteristics (n = 95)	Values
Age at enrollment, median [IQR] (years)	32 [28, 37]
Gravidity, median [IQR]	3 [2, 4]
Antiretroviral treatment (ART) during pregnancy, n (%)	
Dolutegravir-Lamivudine or Emtricitabine-Tenofovir	93 (97.9%)
Dolutegravir-Abacavir-Tenofovir	1 (1.1%)
Efavirenz-Abacavir-Lamivudine	1 (1.1%)
ART exposure during pregnancy, median [IQR] (weeks)	38.6 [36.6, 39.9]
ART initiated prior to conception, n (%)	82 (86.3%)
CD4 count, median [IQR] (cells/mm^3^)	660 [509, 835]^1^
HIV RNA level <400 copies/mL, n (%)	89 (94.7%)^2^
Number of persons in household, median [IQR]	3 [2, 4]^3^
History of TB, n (%)	2 (2.1%)
History of TB preventative therapy (TPT), n (%)	22 (23.2%)
Completion of TPT, n (%)	18 (81.8%)
Household contact with TB in the last 12 months, n (%)	3 (3.2%)^4^
Sibling	1
Cousin	1
Grandmother	1

Median and IQR presented for continuous variables. Counts and percentages presented for categorical variables.

^1^Missing values for CD4 cell count at enrollment: 5; ^2^Missing value for viral load at enrollment: 1; ^3^Missing values for number of persons in the household: 3; ^4^Missing values for household contact with TB: 2.

About three-quarters (72.6%) of PLHIV reported being screened at least once for TB at routine health encounters from pregnancy to two months postpartum. IDCC was the most frequent site of screening (63%), with the second most frequent site of screening occurring at antenatal visits (26%), followed by other (7%) and postpartum visits (4%). None reported positive TB screens during these routine health care encounters. Ninety-three of 95 participants (98%) completed the 2-month postpartum study visit, with nine (10%) participants screening positive for TB symptoms. All nine participants screened positive for cough, with seven participants reporting cough for less than one week, one with cough duration of 1–2 weeks, and one reporting cough duration for greater than two weeks. None reported other TB symptoms.

Eight of 9 maternal participants (89%) with positive TB symptom screen attended the 6-month postpartum visit, with participation in semi-structured interviews. Seven (88%) participants reported attending the clinic for further evaluation of TB symptoms. One participant reported an inability to go to clinic for evaluation due work-related reasons. Sputum samples were collected for four (57%) participants for TB evaluation, one of whom had a chest X-ray (CXR). Five (63%) of the participants reported being tested for COVID-19. None were diagnosed with TB or initiated on TPT. When asked about TB knowledge, five (63%) participants felt well-informed about TB. All participants knew that TB was transmitted in the air. All participants recognized that TB is a treatable disease. Seven participants (88%) reported learning about TB from medical staff.

Nine medical staff participants were recruited for semi-structured interviews from five local government clinics in Gaborone. Among the medical staff, the median age was 38 years [IQR 36, 39] and 67% of the medical staff were female. Four were nurse prescribers, two were nurse midwives, one was a medical officer, one was a nurse, and one was an auxiliary staff member. All medical staff had five years or more practice experience after completing training. Two (22%) had attended a lecture, seminar, or workshop on TB in the last 12 months. All medical staff reported that cough, fever, weight loss, and night sweats were TB symptoms.

**Fig 2:**
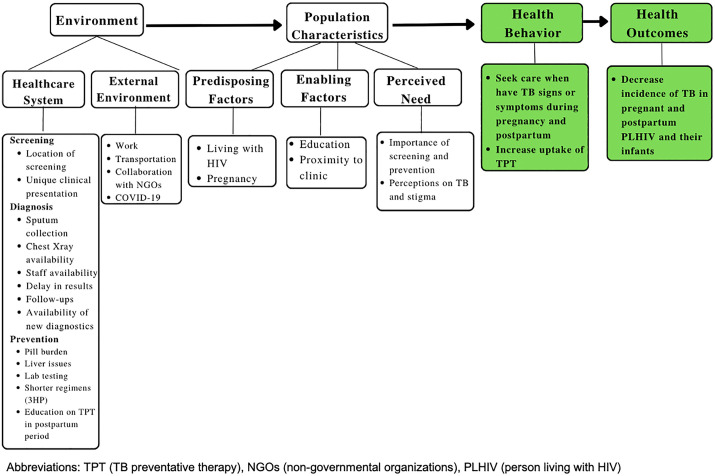
Facilitators and barriers to TB care according to Andersen’s Behavioral Model.

Our analysis of the semi-structured interviews with eight maternal and nine medical staff participants highlighted themes around facilitators and barriers to TB care according to the environment and population levels (**Fig 2**), which inform health behavior and interventions to improve health outcomes for pregnant and postpartum PLHIV.

### Environment

#### Health system.

There were factors in the environment that influenced access to TB screening and care for PLHIV, both in the health system, including screening, diagnosis, and prevention, and the external environment. For TB screening, most maternal participants reported being screened for TB in IDCC, with fewer indicating that TB screening was being performed during their antenatal and postnatal care visits.

“At antenatal, do they ask us about TB? No, I haven’t heard them asking”

[At postnatal]: “They can ask us if we have any TB symptoms, they have never asked me if I have them, I am only asked at IDCC.”

 -Maternal Participant #6

Medical staff participants also identified that while TB screening occurred routinely at IDCC, there was less systematic screening in antenatal and postnatal services.

“As I discovered, we’ve been working on this…in IDCC we don’t face any challenges, because the form or the format of the medical record that we use it compels. But in OPD, I’ll tell you the truth sometimes our clients come with no cards, no medical records, no nothing. You can even miss if this person is pregnant.”

 -Medical Staff Participant #8

Medical staff participants identified that TB screening can be challenging during pregnancy, given that a pregnant person can be asymptomatic, and pregnancy can both mask and mimic TB symptoms, with change in weight being a particularly difficult issue to assess in pregnancy.

“…the signs are not very specific like TB and pregnancy you can have signs in pregnancy that mimic signs in TB because there is generalized weakness, there is shortness of breath because they become a bit anemic also their heart enlarges a bit. So, fatigue, generalized body weakness, shortness of breath, they also have complaint of headache, and then the weight loss. Weight loss can be masked in pregnancy because when you are pregnant, you gain weight.”

 -Medical Staff Participant #9

For TB diagnosis, barriers for maternal participants included challenges with sputum collection, CXR availability, multiple trips to the clinic for evaluation and results, delays in receiving results, and limited staff availability. Only half of the postpartum women referred for symptoms had diagnostic studies performed, including four with sputum sample and one with a CXR.

“As I get to the clinic, they told me that they are going to test me for TB so I was not expecting that they are going to put like a, they used something for me to cough, they were expecting me to cough something has to come out, for me it was tough because I wasn’t having that sputum that they were expecting so I kept on coughing coughing, they were helping me to make it but I ended up making it.”

 -Maternal Participant #1

“Because when I got there, as soon as I arrived, I just sat down by the chairs, then I saw some nurse, we were many, others were going for their own check-ups, so when I saw the nurse, I told her that I came for TB evaluation, the nurse told me that the person who does TB evaluations is not in and there are not sure when the person is coming, I didn’t take long so I went back home.”

 -Maternal Participant #5

Of two maternal participants who were instructed to obtain a CXR, one participant did not receive the CXR imaging study as the equipment was not functioning.

“Yes, I gave them my medical card then they read it. I then told them that I have been referred here. They then asked me if I still have the symptoms then they tested me for COVID-19 and sent me to [clinic 15] to do an X-ray where I found out that the machine is not working.”

 -Maternal Participant #8

Medical staff reported similar challenges related to diagnosis, regarding sputum collection, resources at the clinic (including sputum bottles and reagents), and challenges with follow up.

“At times we have a challenge on patients who are coughing and unable to produce sputum for analysis.”

 -Medical Staff Participant #2

Specific to pregnancy, one medical staff participant reported that it may be risky to send a pregnant woman for CXR given concern for harm to the infant.

“Well pregnancy is a bit risky to send a pregnant woman for an X-ray because it is harmful to the baby. Usually if the patient is coughing and they are able to cough up sputum, we do a sputum test but if not, the ideal test is to do a tuberculin skin test but we do not have that here.”

 -Medical Staff Participant #9

Medical staff shared that new TB diagnostics were being implemented at the clinic level, including urine lipoarabinomannan (LAM), which may aid with the TB diagnostic process and return of results.

“Well, I think the improvement is coming with the TB LAM. If you have a challenge of time or return of results, if you’re saying okay, GeneXpert will take a few days, two to three days to get the result. But still, now we have the TB LAM, you can use it to screen and get the result there and there while the patient is still in the facility, in case the patient wants to relocate somewhere, at least we are able to get the result.”

 -Medical Staff Participant #4

There was limited experience with treatment and management of TB in pregnant and postpartum PLHIV. While none of the maternal participants were diagnosed with TB, only one medical staff provider reported caring for a pregnant patient with TB and HIV, and relayed that TB in pregnancy was considered a special case, requiring consideration of both the health of the mother and that of the infant.

“Apart from the dosing part, there were no challenges, but the concern was the woman’s health plus the baby’s health, so that was the major concern since it was 2 individuals.”

 -Medical Staff Participant #7

Regarding TB prevention, none of the maternal participants were started on TPT after TB evaluation. Yet, medical staff reported the importance of TPT in PLHIV as a tool for prevention. Facilitators to TPT included shorter regimens, such as 3HP, where patients can receive the entire package at once, without need for refills. In addition, medical staff perceptions on TPT can influence uptake of TPT for the patients.

“Today we have got the 3HP. That is the talk of the town…There cannot be any challenges for one reason, my perception, as a clinician, on TPT. It determines the response or the uptake of the client. So, if I explain to them what is this and why is this, then the relationship that I have and the trust they have on me makes everything smooth…I don’t see any [challenges] because in our case, we have them dispense them together with the ARVs…they get here, they get one package and they are gone. They don’t come back for refills and all that. Complete package for the complete period. Unlike yesterday, in the INH [isoniazid] era, you come and INH is out of stock.”

 -Medical Staff Participant #8

Providers noted barriers to TPT, included knowledge gaps, access to baseline liver function testing, pill burden, and breastfeeding during the postpartum period. Some providers knew that TPT was not recommended during pregnancy, but can be given in the postpartum period per Botswana guidelines, while knowledge gaps persisted.

“We use 3HP, which is a combination of Isoniazid and Rifapentine …TPT is taken by every patient living with HIV, excluding pregnant women. Postpartum women can take it…You know TPT…since we are giving it to patients, its challenging for them to take it. They don’t take it because they have heard rumors from other people that it killed their family members and some have liver issues because of it. It’s here and there where you see patients taking it. Even postpartum women don’t take it because they think it will affect their babies since they are breastfeeding.”

 -Medical Staff Participant #1

“Most medical staff does not know about TB prevention treatment and doesn’t know who really qualifies to take it.”

 -Medical Staff Participant #2

#### External environment.

Maternal participants identified work as the main barrier to accessing care at the clinic. One maternal participant was unable to go to clinic for evaluation due to her work schedule. Additional challenges included waiting time and availability of clinic staff, with clinics generally only operating on weekdays and seeing the last patient by noon and without a system allowing for appointments.

“What I can say is, it is tiresome, when you ask for some time off work they come up with sorts of excuses, but the doctor’s appointment is very important. …they would say things like if you don’t come and clean then it won’t be possible, what time are you going to come back because at the hospital there is a long queue, you don’t know the time you are going to be seen or assisted.”

 -Maternal Participant #3

“It was hard for me because I was alone, and when I get to work and ask for day off from the supervisor, he would tell me before you leave make sure you have done something first, so now I will be looking at the time because when you go to the clinic you need to go there early in the morning so by time I want to leave from work I find that the time is already 10 or 11 I end up not going.”

 -Maternal Participant #5

The majority of maternal participants reported that transportation was not a barrier to clinic. One maternal participant reported taking a taxi to protect her child from TB exposure versus using public transportation modes where other persons might present an infection risk to her child.

“I normally take a taxi special to get to the clinic because I don’t want my child to meet, since she is still young so I will just take special going there and back, special is P33 [equivalent to $3 US dollars] going there, P33 coming back home, P33 then I’m done.”

 -Maternal Participant #1

One facilitator to TB diagnosis and contact tracing was collaboration with non-governmental organizations (NGOs) at the local clinics.

“I got help after one [NGO] lady saw me and I told her my story she then told me to wait for her she going to look for someone to help me, she came back with someone to assist me, the person wrote some papers for me then gave me two sample collection bottles for sputum.”

 -Maternal Participant #3

“In our clinic we work with NGOs. So, when a patient is diagnosed with TB, we initiate them on treatment, then they are the ones who do contact tracing.”

 -Medical Staff Participant #1

All maternal participants reported that COVID-19 was not a barrier to accessing care at the clinic. A maternal participant reported that the focus of the evaluations appeared to be COVID-19 during this time, rather than TB.

“Even at antenatal, they are supposed to talk about TB, because they never talk about it, it’s like they sidelined it, ever since COVID-19 came, they only talk COVID-19, not talking about TB, it’s like COVID-19 is at the top of everything.”

 -Maternal Participant #4

Medical staff also reported that there was an identified shift in focus of health care providers towards COVID-19 during the pandemic, which impacted TB care. Medical staff reported that they needed to assess for both COVID-19 and TB due to overlapping symptoms to ensure accurate diagnosis and appropriate management.

“Yes, and COVID-19 is a problem because when it surfaced, symptoms were the same so we started focusing on its symptoms forgetting TB, that’s why now we now believe other people died because of TB not COVID-19 because we were only focusing on COVID-19, but where I was placed that time, we tried to incorporate both of the diseases. When we tested COVID-19, we also tested for TB and this helped since we picked many cases of TB in COVID-19 infected people.”

 -Medical Staff Participant #7

### Population characteristics

#### Predisposing factors.

Maternal participants identified the importance of seeking TB care to prioritize their health and that of their child, which was further influenced by their HIV diagnosis and pregnancy.

“Ever since I was diagnosed with HIV, I accepted my status so when health workers request me to do something I do because that is my well-being.”

 -Maternal Participant #6

“For me to go for check-ups, I wanted to see if I am gaining weight, if I’m eating right, if the baby is growing well. If you just sit at home without going for check-ups, you can [have] complications and deliver with those complications.”

 -Maternal Participant #4

Medical staff participants identified that pregnant and postpartum PLHIV were at high risk of TB disease, with higher risk for TB for those newly diagnosed with HIV in pregnancy and among those who have defaulted on HIV treatment, with lower TB risk among pregnant and postpartum persons on ART with sustained HIV-1 viral suppression.

“It is a problem because firstly HIV lowers CD4, pregnancy as well lowers CD4 count, and all this makes them more vulnerable to contract TB disease.”

 -Medical Staff Participant #2

“But what I have experienced, or what I have realized is that most of the people who are on treatment and are taking their treatment well, we don’t see much of them coming with the TB symptoms.”

 -Medical Staff Participant #4

Medical staff reported follow-up care challenges among postpartum persons due to confinement. In Botswana, confinement represents a period of time that a woman returns to her home village and remains indoors after delivery of a child, usually 90 days in length. The confinement period may make it more difficult to trace their location after delivery.

“I think the only way we can manage is by following them, you see! By making a follow up at their homes. Sometimes they go for confinement elsewhere, they would be gone forever, it would be difficult to follow-up on them. If it was possible, we could be following them like we are doing with HIV positive patients who have defaulted their treatment.”

 -Medical Staff Participant #1

#### Enabling factors.

Maternal and medical staff participants recognized the importance of TB education as an enabling factor to TB care and reported that trainings for TB, particularly on new guidelines, would be helpful for providers working in areas other than IDCC where HIV care is centered, such as antenatal, postpartum care, or sexual reproductive health (SRH).

“I think all they can do is just to keep on teaching pregnant women more about this TB.”

 -Maternal Participant #1

“Here in maternity or SRH, we have challenge of not been trained on new changes on the guidelines. We discover this later and the reason maybe is that we are based on taking care of pregnant mothers and we have been disregarded that there are other services that can be provided except pregnancy.”

 -Medical Staff Participant #2

In addition, medical staff participants found that proximity to clinic and social support for PLHIV were factors that influenced health care usage.

“TB is the leading cause of death in people living with HIV. Those are the reactions that we have. Who is there for them, their proximity to the health facility for easy access to medications and our services.”

 -Medical Staff Participant #8

#### Perceived need.

Maternal participants viewed TB screening and care as important for their health.

“What I can say is, whether I am pregnant or not pregnant I have to screen just to look out for my health, even when I’m negative, I have to screen again after some months.”

 -Maternal participant #2

Some maternal participants perceived TB as on ongoing health problem in their community. Others reported that it was less of a problem, as it is a curable disease that can be treated, unlike HIV.

“There are a lot of TB cases in our community. My cousin had TB it started as pulmonary TB until it developed into bone TB then she died then we all went to test for TB after three months her sister was also diagnosed with TB.”

 -Maternal Participant #4

“No it is not a problem because TB can be healed unlike HIV.”

 -Maternal Participant #6

There were mixed perceptions regarding stigma related to TB among maternal participants, with this being more of a problem in the past than the present.

“Nowadays is okay, but we used to see those people who were having TB they were not treated well they were not living with other people.”

 -Maternal Participant #1

“[Re TB]: Some may discriminate you and some may accept you it just depends.”

 -Maternal Participant #7

Medical staff participants reported the importance of TB screening and prevention in this population, given elevated risk of TB, particularly in those with low CD4 counts. Medical staff generally felt that TB patients were treated well and were given priority in the clinic for evaluation to prevent transmission.

“Well, I think screening is key, because it can’t identify the people who are TB positive, if you don’t screen, yeah. If you strengthen screening, I think you will be able to get all the people who are TB positive. And we’ll be able to initiate them on treatment as soon as possible.”

 -Medical Staff Participant #4

“My perception…I think it’s a good idea, it’s a good initiative because prevention is better than care. And the rate at which HIV…HIV is still, we are still getting new infections…it is difficult to control it, it’s because of that, and TPT is a way to decrease the numbers. I think it is a good initiative.”

 -Medical Staff Participant #9

## Discussion

This mixed methods study aimed to understand TB screening and care practices for pregnant and postpartum PLHIV in Botswana through quantitative and qualitative data collection with key informants, including both maternal and medical staff participants. Our study is unique in that it assessed TB symptoms in the postpartum period, a time period at increased risk of TB [10,11].

Quantitative results on TB screening revealed strengths in TB screening at IDCC visits, with less frequent TB screening in antenatal and postnatal care. Our study found that TB screening occurred most frequently at IDCC-associated HIV health care encounters, which typically occur every three to six months. Screening was less consistent during encounters for antenatal care, with more frequent engagement with the health system, and fewer for six-week postpartum care in our study, despite guidelines that recommend screening at all health care encounters for persons living with HIV. Studies report various experiences with implementation of TB screening during the antenatal and postnatal period. A study on TB case-finding conducted in Eswatini found high screening coverage in antenatal care, but with similar lower coverage in postnatal care consistent with our findings [34]. In this study, only 6 (0.1%) of 5,724 women (both living with HIV and HIV-negative) participants receiving reproductive maternal newborn and child health services who were screened were diagnosed with TB, which may be due to lack of fidelity or limited sensitivity of symptom screening [34]. A study in Kenya found that integrating TB services in postnatal care was acceptable and feasible, but there was a low number of cases detected [18]. Another study in Kenya evaluated integration of TB screening into prevention of mother-to-child transmission programs, with a third (33%) of postpartum PLHIV found to have a positive WHO TB symptom screen at infant immunization visits [21].

Opportunities to improve implementation of TB screening include intensifying screening for TB as part of antenatal care, as scheduled encounters with the health care system during this period occur more frequently than encounters for HIV care among PLHIV with sustained viral suppression. While there are less frequent routine care encounters during the postpartum period, with generally 1 visit at 6 weeks postpartum, it is a key opportunity for close monitoring of postpartum PLHIV. While TB symptoms may be masked in pregnancy, TB symptoms can be exacerbated in the postpartum period due to immune changes after delivery [1]. There are also unique cultural considerations in the postpartum period to consider, such as confinement, depending on the context. Other venues for integration of structured TB screening for women of reproductive age living with HIV include sexual reproductive health clinics and well child/child immunization visits. In IDCC, there is a required form prompting the care provider to screen patients for TB symptoms and document results. This form is not used in other departments, such as outpatient departments, antenatal, or postpartum clinics. The success of a structured and required TB screening form in use in Botswana’s HIV clinics could be adopted in these settings, with adequate staff training on TB screening practices and TB referral pathways.

Qualitative analysis according to the Andersen’s Behavioral Model (ABM) revealed factors influencing access to TB care at the environmental and population levels. Prior studies have applied ABM to evaluate barriers and facilitators to access healthcare in global health settings [35,36]. In our study, maternal participants cited motivations for TB evaluation, such as prioritizing their own health and the health of their pregnancy to optimize the health of their infant, but faced barriers such as work commitments and clinic wait times. Medical staff highlighted vulnerability among PLHIV, identified knowledge gaps among medical staff, and stressed the importance of TB screening, diagnosis, and preventative care. The timing of our study is important, as it was performed during the COVID-19 pandemic, at which time many of the study participants reported being evaluated for COVID-19 when referred for evaluation of TB. While it did not impact health-seeking behaviors for maternal participants, there was a shift in focus away from TB toward COVID-19 for medical staff, which impacted routine TB screening and care practices, a tradeoff reported in other studies, with need for ongoing analysis as the COVID-19 pandemic evolves [37,38].

The findings of this study align with previous research that demonstrate barriers to TB care, particularly given long and complex pathways for TB diagnosis [39–41]. Our study additionally demonstrated a diagnostic gap for TB evaluation, as only half of the postpartum women with positive symptomatic screening were asked to provide a sputum sample as part of their evaluation and only one participant received a chest radiograph. There were additionally structural barriers to diagnosis, including procedural barriers to sputum production, CXR machine availability and functionality, and knowledge barriers regarding CXR use in pregnant persons. Other studies have reported similar barriers to TB diagnosis in low- and middle-income countries, including sputum sample collection, lack of resources such as diagnostic supplies, and shortage of healthcare personnel [42,43]. One other diagnostic barrier could be that symptoms may have resolved once the maternal participants presented to the clinic for evaluation. Newer diagnostic tests and algorithms, including GeneXpert Ultra and urine LAM assays, may improve the sensitivity of detecting TB disease for PLHIV, particularly given concern for paucibacillary disease [44–46].

Our study also highlights gaps that exist regarding uptake of TPT for PLHIV, with only 23.2% of participants who reported a history of TPT, which is likely an underestimation as TPT may have been started postpartum after our follow-up. There was also limited medical staff knowledge of TPT recommendations, particularly for postpartum PLHIV. A recent review found gaps in specific screening and TPT recommendations for pregnant and breastfeeding PLHIV in national guidelines from countries with President’s Emergency Plan of AIDS Relief (PEPFAR) support, with need for further guidance on implementation of the guidelines that are available in programmatic settings [47]. In addition, most countries recommend isoniazid-based TPT regimens for pregnant and breastfeeding PLHIV, with some recommending isoniazid combined with rifampicin (3RH) or rifapentine (3HP), with need for larger studies on this regimen in pregnancy [47,48]. As Botswana guidelines recommend TPT for PLHIV in the postpartum period, there are opportunities to increase and monitor uptake of TPT, particularly with the implementation of shorter regimens, such as 3HP.

Our study did not identify distance or travel as a barrier to accessing TB care, which is in contrast to a study that did find transportation and distance to be a barrier to TB active case-finding in low and middle income countries [42]. In Botswana, there is unique design of the government health system, as there is a health post or clinic strategically placed within 15 kilometers of all communities, which may explain this result. Our study did not have any participants diagnosed with TB, which may reflect the health of our maternal participants, as all were on ART treatment and 95% had achieved HIV viral suppression at enrollment. However, the lack of diagnosis of TB may reflect limitations of symptom screening, with need for improved screening approaches for this population [20,49]. A recent systematic review commissioned by the WHO on TB and pregnancy reported a low pooled sensitivity (38%) of symptom-based screening among pregnant PLHIV [4]. Despite screening limitations, medical staff should remain vigilant in screening for TB in pregnant and postpartum PLHIV, even when they are adherent to ART therapy with adequate CD4 counts. A recent study in India showed that PLHIV with adequate CD4 counts express less interferon-gamma from pregnancy through the postpartum period compared to persons without HIV, with increased risk of postpartum TB disease [50].

While this study offers many valuable insights, it does have its limitations. Participants self-reported whether they were screened for TB at routine encounters and were enrolled at various times during the pregnant and postpartum period, which presents the risk of recall bias. Among participants who screened positive for TB symptoms and were referred to their local clinic, participants self-reported diagnostics and the evaluation that was performed, which may depend on their understanding of the health visit. However, maternal participant medical records were also reviewed in an effort to corroborate participant self-reporting. Medical staff were recruited via purposive sampling from local health facilities, which only captures perspectives from a small sample of medical staff working in an urban area. There may be limited generalizability to rural settings or settings with lower HIV and TB prevalence. While our study design allowed for qualitative interviews with up to 10 maternal participants and 10 medical staff participants [51], thematic saturation was achieved with our sample size of 8 maternal participants and 9 medical staff members.

### Future directions

There remain opportunities to improve and intensify current TB screening and care practices for pregnant and postpartum PLHIV. Importantly, TB disease incidence and prevalence data is urgently needed for the pregnant and postpartum periods among PLHIV. Additional opportunities include education on TB care for pregnant and postpartum PLHIV and medical staff providers in clinics that routinely engage with this population. Future research is needed to improve TB care for pregnant and postpartum PLHIV, including adapted screening algorithms, optimal timing and implementation of diagnostic tests, shorter treatment regimens, and effective preventative strategies, including uptake of TPT and inclusion in trials for TB vaccines [4,20,52–54].

## Conclusion

This study highlights the complexities and challenges associated with TB screening, diagnosis, and prevention among pregnant and postpartum PLHIV in Botswana. Further studies are needed to develop and evaluate interventions to improve and support TB screening and care delivery for pregnant and postpartum PLHIV, recognizing that improvement in the health of pregnant women optimizes pregnancy outcomes and the health of their infants.

## Supporting information

S1 Data Data. (XLSX)

## References

[1] Mathad JS, Gupta A. Tuberculosis in pregnant and postpartum women: epidemiology, management, and research gaps. Clin Infect Dis. 2012;55(11):1532–49. 10.1093/cid/cis732 22942202 PMC3491857

[2] Sugarman J, Colvin C, Moran AC, Oxlade O. Tuberculosis in pregnancy: an estimate of the global burden of disease. Lancet Glob Health. 2014;2(12):e710–6. doi: 10.1016/S2214-109X(14)70330-4 25433626

[3] World Health Organization. Global Tuberculosis Report 2024. [Internet]. 2024 [cited 2024 Nov 15]. Available from: https://www.who.int/teams/global-programme-on-tuberculosis-and-lung-health/tb-reports/global-tuberculosis-report-2024

[4] World Health Organization. Optimal and early inclusion of pregnant and lactating women in tuberculosis research: consensus statement. 2025.

[5] Getahun H, Sculier D, Sismanidis C, Grzemska M, Raviglione M. Prevention, diagnosis, and treatment of tuberculosis in children and mothers: evidence for action for maternal, neonatal, and child health services. J Infect Dis. 2012;205 Suppl 2:S216–27. doi: 10.1093/infdis/jis009 22448018

[6] Gupta A, Bhosale R, Kinikar A, Gupte N, Bharadwaj R, Kagal A, et al. Maternal tuberculosis: a risk factor for mother-to-child transmission of human immunodeficiency virus. J Infect Dis. 2011;203(3):358–63. doi: 10.1093/infdis/jiq064 21208928 PMC3071111

[7] Salazar-Austin N, Hoffmann J, Cohn S, Mashabela F, Waja Z, Lala S, et al. Poor obstetric and infant outcomes in human immunodeficiency virus-infected pregnant women with tuberculosis in South Africa: The Tshepiso Study. Clin Infect Dis. 2018;66(6):921–9. doi: 10.1093/cid/cix851 29028970 PMC5849996

[8] World Health Organization. Roadmap towards ending TB in children and adolescents: Third edition. 2023. [cited 2024 Nov 15]; Available from: https://www.who.int/publications/i/item/9789240084254

[9] Yilma A, Bailey H, Karakousis PC, Karanika S. HIV/tuberculosis coinfection in pregnancy and the postpartum period. J Clin Med. 2023;12(19):6302. doi: 10.3390/jcm12196302 37834946 PMC10573401

[10] Zenner D, Kruijshaar ME, Andrews N, Abubakar I. Risk of tuberculosis in pregnancy: a national, primary care-based cohort and self-controlled case series study. Am J Respir Crit Care Med. 2012;185(7):779–84. doi: 10.1164/rccm.201106-1083OC 22161161

[11] Gupta A, Nayak U, Ram M, Bhosale R, Patil S, Basavraj A, et al. Postpartum tuberculosis incidence and mortality among HIV-infected women and their infants in Pune, India, 2002-2005. Clin Infect Dis. 2007;45(2):241–9. doi: 10.1086/518974 17578786

[12] Saha A, Escuduero J, Layouni T, Richardson B, Hou S, Mugo N, et al. Mycobacterium tuberculosis-specific T-cell responses are impaired during late pregnancy with elevated biomarkers of tuberculosis risk postpartum. J Infect Dis. 2022;225(9):1663–74. doi: 10.1093/infdis/jiab614 34929030 PMC9071276

[13] World Health Organization. WHO recommendations on antenatal care for a positive pregnancy experience: screening, diagnosis and treatment of tuberculosis disease in pregnant women [Internet]. 2016 [cited 2025 Jan 15]. Available from: https://www.who.int/publications/i/item/9789241549912

[14] World Health Organization. TB and pregnancy. 2024 [cited 2025 Jan 13]; Available from: https://www.who.int/teams/global-tuberculosis-programme/tb-reports/global-tuberculosis-report-2024/featured-topics/tb-and-pregnancy#refs

[15] World Health Organization. WHO consolidated guidelines on tuberculosis. Module 6: tuberculosis and comorbidities. [Internet]. 2024 [cited 2024 Oct 15]. Available from: https://www.who.int/publications/i/item/9789240087002 38713785

[16] Snow KJ, Bekker A, Huang GK, Graham SM. Tuberculosis in pregnant women and neonates: a meta-review of current evidence. Paediatr Respir Rev. 2020;36:27–32. doi: 10.1016/j.prrv.2020.02.001 32144052

[17] Uwimana J, Jackson D. Integration of tuberculosis and prevention of mother-to-child transmission of HIV programmes in South Africa. Int J Tuberc Lung Dis. 2013;17(10):1285–90. doi: 10.5588/ijtld.12.0068 24025379

[18] Ndwiga C, Birungi H, Undie C-C, Weyenga H, Sitienei J. Feasibility and effect of integrating tuberculosis screening and detection in postnatal care services: an operations research study. BMC Health Serv Res. 2013;13:99. doi: 10.1186/1472-6963-13-99 23496997 PMC3602180

[19] Kali PBN, Gray GE, Violari A, Chaisson RE, McIntyre JA, Martinson NA. Combining PMTCT with active case finding for tuberculosis. J Acquir Immune Defic Syndr. 2006;42(3):379–81. doi: 10.1097/01.qai.0000218434.20404.9c 16645548

[20] LaCourse SM, Cranmer LM, Matemo D, Kinuthia J, Richardson BA, John-Stewart G, et al. Tuberculosis case finding in HIV-infected pregnant women in kenya reveals poor performance of symptom screening and rapid diagnostic tests. J Acquir Immune Defic Syndr. 2016;71(2):219–27. doi: 10.1097/QAI.0000000000000826 26334736 PMC4712112

[21] Cranmer LM, Langat A, Ronen K, McGrath CJ, LaCourse S, Pintye J, et al. Integrating tuberculosis screening in Kenyan Prevention of Mother-To-Child Transmission programs. Int J Tuberc Lung Dis. 2017;21(3):256–62. doi: 10.5588/ijtld.16.0478 28225335 PMC5729039

[22] Hamda SG, Tshikuka JG, Joel D, Setlhare V, Monamodi G, Mbeha B, et al. Contribution of Xpert® MTB/RIF to tuberculosis case finding among pregnant women in Botswana. Public Health Action. 2020;10(2):76–81. doi: 10.5588/pha.19.0077 32639478 PMC7316435

[23] Daftary A, Furin J, Zelnick JR, Venkatesan N, Steingart K, Smelyanskaya M, et al. TB and women: a call to action. Int J Tuberc Lung Dis. 2020;24(12):1312–5. doi: 10.5588/ijtld.20.0414 33317679

[24] Simpson G, Philip M, Vogel JP, Scoullar MJL, Graham SM, Wilson AN. The clinical presentation and detection of tuberculosis during pregnancy and in the postpartum period in low- and middle-income countries: a systematic review and meta-analysis. PLOS Glob Public Health. 2023;3(8):e0002222. doi: 10.1371/journal.pgph.0002222 37611006 PMC10446195

[25] Narh-Bana SA, Kawonga M, Odopey SA, Bonsu F, Ibisomi L, Chirwa TF. Factors influencing the implementation of TB screening among PLHIV in selected HIV clinics in Ghana: a qualitative study. BMC Health Serv Res. 2022;22(1):898. doi: 10.1186/s12913-022-08295-6 35818070 PMC9272598

[26] Resende NHD, Martins UCDM, Ramalho-de-Oliveira D, Silva DID, de Miranda SS, Reis AMM, et al. The medication experience of TB/HIV coinfected patients: qualitative study. Int J Environ Res Public Health. 2022;19(22):15153. doi: 10.3390/ijerph192215153 36429870 PMC9690802

[27] Khoza L, Mulondo S, Lebese R. Perspectives on pregnant women’s educational needs to prevent TB complications during pregnancy and the neonatal period: a qualitative study. BMC Public Health. 2023;23(1):1997. 10.1186/s12889-023-16770-w 37833655 PMC10576336

[28] UNAIDS. Botswana 2023. [cited 2025 Jan 13]; Available from: https://aidsinfo.unaids.org/

[29] Ministry of Health and Wellness. Botswana National Tuberculosis Programme Manual: 8th edition. 2018.

[30] Ministry of Health and Wellness, Republic of Botswana. The Botswana 2023 Integrated HIV Clinical Care Guidelines [Internet]. 2023 [cited 2024 Dec 15]. Available from: https://www.moh.gov.bw/Publications/HIV_treatment_guidelines.pdf

[31] Botswana Ministry of Health. Tuberculosis Preventative Therapy Handbook. 2022.

[32] Fetters MD, Curry LA, Creswell JW. Achieving integration in mixed methods designs-principles and practices. Health Serv Res. 2013;48(6 Pt 2):2134–56. doi: 10.1111/1475-6773.12117 24279835 PMC4097839

[33] Andersen RM. Revisiting the behavioral model and access to medical care: does it matter? J Health Soc Behav. 1995;36(1):1–10. doi: 10.2307/2137284 7738325

[34] Hartsough K, Teasdale CA, Shongwe S, Geller A, Pimentel De Gusmao E, Dlamini P, et al. Enhanced Integration of TB Services in Reproductive Maternal Newborn and Child Health (RMNCH) Settings in Eswatini. PLOS Glob Public Health. 2022;2(4):e0000217. doi: 10.1371/journal.pgph.0000217 36962173 PMC10021747

[35] Holtzman CW, Shea JA, Glanz K, Jacobs LM, Gross R, Hines J, et al. Mapping patient-identified barriers and facilitators to retention in HIV care and antiretroviral therapy adherence to Andersen’s Behavioral Model. AIDS Care. 2015;27(7):817–28. doi: 10.1080/09540121.2015.1009362 25671515 PMC4400221

[36] Sundararajan R, Alakiu R, Ponticiello M, Birch G, Kisigo G, Okello E, et al. Understanding traditional healer utilisation for hypertension care using the Andersen model: a qualitative study in Mwanza, Tanzania. Glob Public Health. 2023;18(1):2191687. doi: 10.1080/17441692.2023.2191687 36973183 PMC10065353

[37] Pai M, Kasaeva T, Swaminathan S. Covid-19’s devastating effect on tuberculosis care— a path to recovery. N Engl J Med. 2022;386(16). 10.1056/NEJMp2118145 34986295

[38] Dheda K, Perumal T, Moultrie H, Perumal R, Esmail A, Scott AJ, et al. The intersecting pandemics of tuberculosis and COVID-19: population-level and patient-level impact, clinical presentation, and corrective interventions. Lancet Respir Med. 2022;10(6):603–22. doi: 10.1016/S2213-2600(22)00092-3 35338841 PMC8942481

[39] Kerkhoff AD, Kagujje M, Nyangu S, Mateyo K, Sanjase N, Chilukutu L, et al. Pathways to care and preferences for improving tuberculosis services among tuberculosis patients in Zambia: a discrete choice experiment. PLoS One. 2021;16(8):e0252095. doi: 10.1371/journal.pone.0252095 34464392 PMC8407587

[40] De Schacht C, Mutaquiha C, Faria F, Castro G, Manaca N, Manhiça I, et al. Barriers to access and adherence to tuberculosis services, as perceived by patients: a qualitative study in Mozambique. PLoS One. 2019;14(7):e0219470. doi: 10.1371/journal.pone.0219470 31291352 PMC6619801

[41] Krishnan L, Akande T, Shankar AV, McIntire KN, Gounder CR, Gupta A, et al. Gender-related barriers and delays in accessing tuberculosis diagnostic and treatment services: a systematic review of qualitative studies. Tuberc Res Treat. 2014;2014:215059. doi: 10.1155/2014/215059 24900921 PMC4037602

[42] Fenta MD, Ogundijo OA, Warsame AAA, Belay AG. Facilitators and barriers to tuberculosis active case findings in low- and middle-income countries: a systematic review of qualitative research. BMC Infect Dis. 2023;23(1):515. doi: 10.1186/s12879-023-08502-7 37550614 PMC10405492

[43] Andom AT, Gilbert HN, Ndayizigiye M, Mukherjee JS, Lively CT, Nthunya J, et al. Understanding barriers to tuberculosis diagnosis and treatment completion in a low-resource setting: A mixed-methods study in the Kingdom of Lesotho. PLoS One. 2023;18(5):e0285774. doi: 10.1371/journal.pone.0285774 37167298 PMC10174523

[44] World Health Organization. WHO operational handbook on tuberculosis. Module 3: diagnosis – rapid diagnostics for tuberculosis detection. 3rd ed. 2024. 38527162

[45] Dorman SE, Schumacher SG, Alland D, Nabeta P, Armstrong DT, King B, et al. Xpert MTB/RIF Ultra for detection of Mycobacterium tuberculosis and rifampicin resistance: a prospective multicentre diagnostic accuracy study. Lancet Infect Dis. 2018;18(1):76–84. doi: 10.1016/S1473-3099(17)30691-6 29198911 PMC6168783

[46] Peter JG, Zijenah LS, Chanda D, Clowes P, Lesosky M, Gina P, et al. Effect on mortality of point-of-care, urine-based lipoarabinomannan testing to guide tuberculosis treatment initiation in HIV-positive hospital inpatients: a pragmatic, parallel-group, multicountry, open-label, randomised controlled trial. Lancet. 2016;387(10024):1187–97. doi: 10.1016/S0140-6736(15)01092-2 26970721

[47] Hirsch-Moverman Y, Hsu A, Abrams EJ, Killam WP, Moore B, Howard AA. Guidelines for tuberculosis screening and preventive treatment among pregnant and breastfeeding women living with HIV in PEPFAR-supported countries. PLoS One. 2024;19(4):e0296993. doi: 10.1371/journal.pone.0296993 38625930 PMC11021021

[48] Mathad JS, Savic R, Britto P, Jayachandran P, Wiesner L, Montepiedra G, et al. Pharmacokinetics and safety of 3 months of weekly rifapentine and isoniazid for tuberculosis prevention in pregnant women. Clin Infect Dis. 2022;74(9):1604–13. doi: 10.1093/cid/ciab665 34323955 PMC9070820

[49] Hoffmann CJ, Variava E, Rakgokong M, Masonoke K, van der Watt M, Chaisson RE, et al. High prevalence of pulmonary tuberculosis but low sensitivity of symptom screening among HIV-infected pregnant women in South Africa. PLoS One. 2013;8(4):e62211. doi: 10.1371/journal.pone.0062211 23614037 PMC3629105

[50] Bhosale R, Alexander M, Deshpande P, Kulkarni V, Gupte N, Gupta A, et al. Stages of pregnancy and HIV affect diagnosis of tuberculosis infection and Mycobacterium tuberculosis (MTB)-induced immune response: findings from PRACHITi, a cohort study in Pune, India. Int J Infect Dis. 2021;112:205–11. doi: 10.1016/j.ijid.2021.09.010 34517050 PMC8715310

[51] Hennink MM, Kaiser BN, Marconi VC. Code saturation versus meaning saturation: how many interviews are enough? Qual Health Res. 2017;27(4):591–608. 10.1177/1049732316665344 27670770 PMC9359070

[52] Jones AJ, Mathad JS, Dooley KE, Eke AC. Evidence for implementation: management of TB in HIV and pregnancy. Curr HIV/AIDS Rep. 2022;19(6):455–70. doi: 10.1007/s11904-022-00641-x 36308580 PMC9617238

[53] Maugans C, Loveday M, Hlangu S, Waitt C, Van Schalkwyk M, van de Water B, et al. Best practices for the care of pregnant people living with TB. Int J Tuberc Lung Dis. 2023;27(5):357–66. doi: 10.5588/ijtld.23.0031 37143222 PMC10171489

[54] Sumner T, Clark RA, Prys-Jones TO, Bakker R, Churchyard G, White RG. The potential impact of new tuberculosis vaccines on the burden of tuberculosis in people with HIV in South Africa. AIDS. 2025;39(2):175–83. https://10.1097/QAD.0000000000004038 39411889 10.1097/QAD.0000000000004038 PMC11676631

